# Dr. Roberto Miguel Klein Herbarium (FURB), Blumenau, Southern Brazil

**DOI:** 10.3897/phytokeys.42.6865

**Published:** 2014-10-13

**Authors:** André Luís de Gasper, Alexander Christian Vibrans, Luís Adriano Funez, Morilo José Rigon-Jr, Felipe Bittencourt, Carina Vieira

**Affiliations:** 1 Herbarium Curator; 2 Universidade Regional de Blumenau (University of Blumenau). Antônio da Veiga, 140 - Victor Konder. CEP: 89012-900 - Blumenau – Santa Catarina- Brasil

**Keywords:** Catalogue, Scientific collection, Regional University of Blumenau, Ferns, Vascular plants, Spermatophyta, Pteridophyta, Compositae, Leguminosae, Southern Brazil, Rain Forest, Evergreen Rainforest, National Parks, Biodiversity

## Abstract

The premise of this study is to present the collection of the FURB herbarium, its collection area and type specimens, as well as its projects and contributions to the flora of the Subtropical Atlantic Forest. The FURB herbarium currently has nearly 41,000 records of vascular plants and has the largest collection of lycophytes and ferns in Southern Brazil, with more than 8,000 records. More than 4,500 scanned images of 4,436 species are available online, and it is expected that the whole collection will be scanned in less than one year. There are 198 families of angiosperms, 33 of ferns, three of lycophytes and six of gymnosperms. All collections of the Floristic and Forest Inventory of Santa Catarina project are recorded in FURB, which represents almost 35,000 herbarium specimens. The families with the largest number of species are: Cyperaceae (109 species), Rubiaceae (129), Solanaceae (131), Poaceae (155), Melastomataceae (157), Myrtaceae (257), Orchidaceae (288), Fabaceae (323), and Asteraceae (426), between angiosperms. Among the ferns and lycophytes are: Hymenophyllaceae (30), Thelypteridaceae (31), Aspleniaceae (32), Dryopteridaceae (43), Pteridaceae (54) and Polypodiaceae (60). There are five type specimens among them: one holotype, one isotype and three paratypes. To date, the FURB herbarium has donated 19,521 herbarium duplicates for identification or expansion of other herbaria.

## Introduction

The Dr. Roberto Miguel Klein Herbarium (FURB) was founded in 1990 by Lucia Sevegnani, PhD. Roberto Miguel Klein (1923-1992) was an important botanist and ecologist. In 1949, together with Father Raulino Reitz, he founded the Sellowia Journal, which published contributions of researchers linked to Herbário Barbosa Rodrigues (HBR). In addition to HBR, the Sellowia papers are considered Klein’s major works, among those that treated the relation between malaria outbreaks and the local flora (Klein 1967) and addressed the vegetation structure of Southern Brazil, published in a series of papers co-authored by Henrique Pimenta Veloso (Veloso and Klein 1957, 1959, 1961, 1963, 1968a, 1968b).

Klein and Reitz also created the Flora Ilustrada Catarinense (Reitz 1965; Citadini-Zanette 2013), published in 149 fascicles between 1965 and 1989. In addition to conducting a floristic assessment of Santa Catarina State, Klein studied autoecology and gathered information about tree communities, making himself one of the outstanding dendrologists of Southern Brazil (Amado 2013). His importance has been proven by tributes such as the genus *Kleinodendron* L.B.Sm. & Downs, besides 75 specific epithets, from 37 families (IPNI 2012). After his death, Klein’s scientific contributions were not forgotten. The Flora Ilustrada Catarinense came to be published from 1996 on, with 17 volumes published to date (Bortoluzzi et al. 2011).

The FURB herbarium has been registered in Index Herbariorum since 2005, under the acronym FURB (http://sweetgum.nybg.org/ih/herbarium.php?irn=148203). The aim of the herbarium is to study the plant diversity of the local vegetation, especially that of the Serra do Itajaí National Park, Itajaí Valley, and Santa Catarina State. Initially the collection was established by professors for didactic purposes, but with the advent of new projects, such as the Floristic and Forest Inventory of Santa Catarina (IFFSC project - Vibrans et al. 2010), the collection has undergone a massive increase in the amount of material. At the beginning of the IFFSC project, the FURB herbarium had only approximately 5,000 herbarium specimens (now there are more than 41,000). At present, 86% of the FURB collection is georeferenced, with only a few early specimens not having coordinates.

The software that is used has been developed especially for the herbarium and the data has become available online, in INCT databases (http://inct.splink.org.br/), SpeciesLink (http://splink.cria.org.br/), and Reflora (http://reflora.jbrj.gov.br). The software (Herbaria 3.1), an Access-based software, has been developed by EPAGRI (http://www.epagri.sc.gov.br) in partnership with the herbarium staff (for more information see Miszinski et al. 2012).

The angiosperm collections are organized according to the APG system (Reveal and Chase 2011), the ferns according to Rothfels et al. (2012) and Smith et al. (2006), the lycophytes according to Øllgaard (2012), and the gymnosperms according to Christenhusz et al. (2011).

## Data resources

The data underpinning the analyses reported in this paper are deposited in the GBIF, the Global Biodiversity Information Facility, http://ipt.pensoft.net/ipt/resource.do?r=furb_herbarium_database_11-09-2014

## Available data

**Figure 1.**
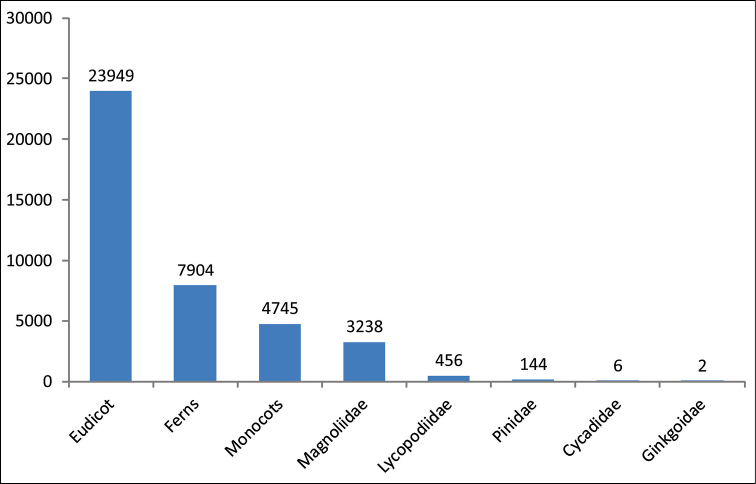
Number of specimens distributed among the division of vascular plants.

**Figure 2.**
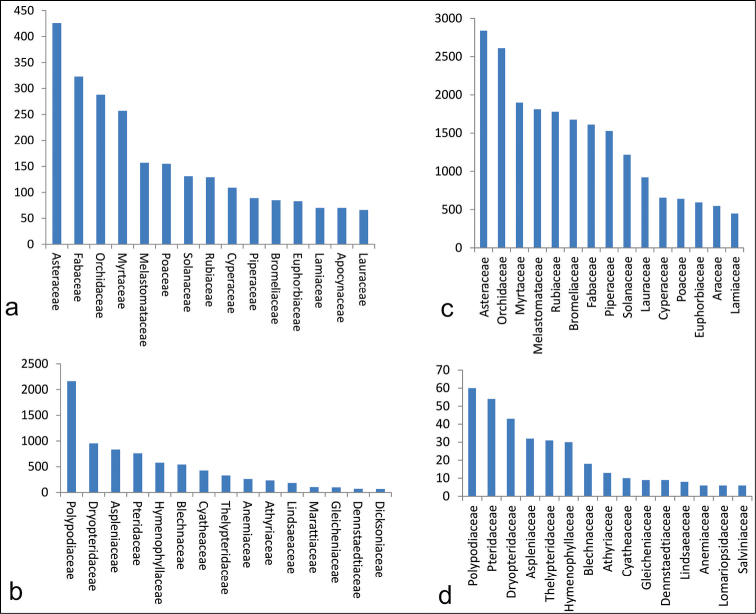
Number of specimens of the main families of angiosperms (**a**), ferns and lycophytes (**b**) in FURB collection. Number of specimens of angiosperms (**c**), ferns and lycophytes (**d**).

The herbarium has 41,325 specimens (Figure 1 and Figure 2), in addition to the largest collection of ferns and lycophytes (8,360 specimens) in Santa Catarina State. The full database is available via the INCT Virtual Herbarium (http://inct.splink.org.br/FURB). There are 4,556 images of 4,436 species.

## Recent projects

### Flora Catarinense Revisited

This project aims to find the nomenclatural types collected in Santa Catarina in herbaria and publications; recollect specimens in the field in the type localities, mainly the older ones; scan nomenclatural types; perform collections; and get information about rare and endemic species that are only known by the type or by very few documented specimens in collections.

### IFFSC – Floristic and Forest Inventory of Santa Catarina State

This project aims to develop the floristic inventory of forest formations in Santa Catarina and generate information to support the formulation of forest policy in the state, in particular the economic-ecological zoning for rural land use planning and environmental licensing, as well as the defining of priority areas for conservation, restoration of degraded ecosystems, and the updating of red lists. To complete these objectives, the remaining forests are periodically inventoried and their horizontal and vertical structures analyzed. The genetic diversity and structure of populations of 13 endangered species are assessed, as well as the social and economic importance of the state’s native forest resources. Finally, a georeferenced information system has been designed and implemented and is to be updated in order to assist the preparation of the endangered species list. The project publications can be downloaded from the IFFSC website (http://www.iff.sc.gov.br/ – Vibrans et al. 2013a, 2013b, 2013c, 2012a, 2012b).

### DNA bank

The establishment of a DNA database linked to the FURB herbarium aims to provide subsidies for studies in molecular biology including species of flora of Santa Catarina. In Brazil these initiatives have been shown to be necessary, since, despite the enormous biodiversity of the flora of the country, molecular studies are scarce (Santos et al. 2002). The bank starts in 2014 with the goal of maintaining samples of the main species of Santa Catarina’s vascular plants and fungi. Field surveys will be performed and samples collected from young leaves will be stored in silica gel. This inexpensive andeffective method, neither injurious to the DNA nor detrimental to the dehydration of leaves (Chase and Hills 1991), grants researchers the ability to preserve the DNA material for at least 10 years (Hodkinson et al. 2007), reducing the costs of research and allowing material to be donated and loaned. Gaudeul and Rouhan (2013) recommend a universalization of the method and this should be done in any field, since it is practical, avoids surveys in specific fields for this purpose, and increases the scientific value of the specimens and collection. In fact, other field surveys will be done, in addition to those related to the project, to ensure the existence of samples in DNA database. In the first year, the collections will prioritize the Itajaí Valley region of the state, which is home to 54 municipalities, and it estimated that at least 20% of the tree species will be collected and made ​​available for study. The sample for the DNA bank will continue with the second cycle of IFFSC, whose collections will be performed throughout the state, and it is estimated that material will be collected for DNA extraction for about 75% of the tree species. The database of the species can be found at www.furb.br/herbarium.

## Temporal coverage

**Figure 3.**
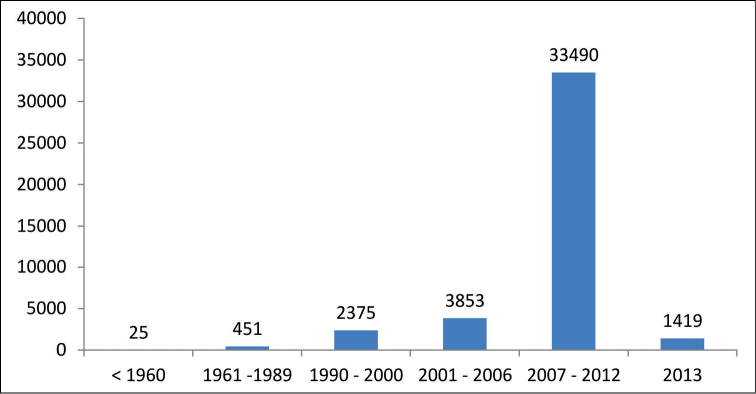
Temporal coverage of Herbarium FURB.

The earliest specimen is dated March 1, 1937, a *Prosopis nigra* Hieron. (Fabaceae), but the first specimen for the FURB herbarium was collected on September 7, 1991, of a *Plinia rivularis* (Cambess.) Rotman. (Myrtaceae). The distribution of collected specimens by year can be seen in Figure 3. A great increase occurred between 2007 and 2012, during the first cycle of the IFFSC project. The second cycle of the project will start in 2014 and will surely result in an increase of the the number of specimens.

## Plant sampling

**Figure 4.**
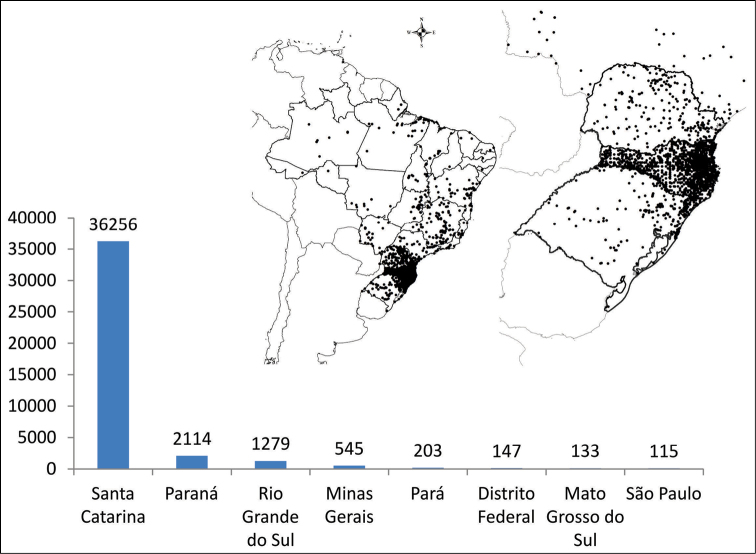
Spatial coverage of FURB collection. Only the hundred best states collected.

Most of the plant samples are from Southern Brazil, Santa Catarina State (Figure 4) being the most widely represented area, and the aim is to cover the widest possible range of plant diversity of this territory. The majority of the samples comes from the IFFSC project and from the field work of undergraduate students. Other parts of the Brazilian South are represented mainly by exchanged samples.

### Plant processing procedures

After being dried in ovens at 65 to 70 degrees Celsius, the specimens are incorporated into the database. All data such as scientific name, location, city, coordinates, altitude, and general descriptions of the plant are recorded. The registration number is generated automatically by the software. Afterwards, the plants are glued with hot glue onto A2 sheets, packed in plastic bags in order to avoid being contaminated by insects or fungi and to prevent loss of material during handling, and subsequently frozen for five days at -20 degrees Celsius. Next, the plants are stored in specific cans that are sealed and stored separately in groups: lycophytes and ferns, gymnosperms and angiosperms. Among the groups, the cans are alphabetized according to family and gender.

All databases are reviewed by a DataClean tool, from CRIA (http://splink.cria.org.br/dc/index?criaLANG=pt&colecao=FURB), which checks the coordinates, toponymy, scientific names, and their authors. The “doubtful” records are reviewed by the curator. The names are checked using the List of Species of Brazilian Flora (2014), and the identifications are made by specialists visiting the herbarium or by comparison with photos or duplicates sent to other institutions.

The FURB herbarium uses a specific software, Herbaria 3.1 (Miszinski et al. 2012), that includes several control possibilities of the collection. These control possibilities include information about the synonymous names used, preventing the same species from being registered under several names. Thus, we use specific fields for family, genus, and specific epithet. When we enter a genus name, its family and division is automatically filled based on an ancillary table. A list of epithets linked to the genus is then released, allowing the choice of the same (new epithets can be linked at any time), which is chosen to fill the author’s name and still shows the presence of varieties or subspecies.

## Rare, endemic species and ecosystems sampled

FURB maintains 68% of all species listed by the List of Species of Brazilian Flora (2014) for Santa Catarina and 33.9% of all species in the Brazilian Atlantic Forest (Stehmann et al. 2009). The Atlantic Forest in Santa Catarina has three forest types: Evergreen Rainforest, Mixed Forest, and Seasonal Forest (for more information see Klein 1978 and Oliveira-Filho et al. 2013). 75% of FURB samples were collected in Evergreen Rainforest, 16% in Mixed Forest, and 5% in Seasonal Forest. The other formations (grasslands, mangroves, and coastal vegetation) constitutes 4% of the collections. Species of natural grasslands are represented still less, but will increase due to sample campaigns to be held within the IFFSC project during 2014 and 2015. Furthermore, 27 species of the Brazilian Official List of Endangered Species (Brazil 2008) are represented in the collection, available for conservation and other studies, 19 of these being native of Santa Catarina. Of the 235 endemic species of Santa Catarina (Brazilian List of Species of Flora 2014), 52 are found in the herbarium.

The database SpeciesLink (http://splink.cria.org.br/) shows that the FURB herbarium possesses 0.0725 records per sq km of the Southern region. Although seemingly small, it is a high number compared to other featured herbaria in Santa Catarina; FLOR, JOI and CRI have 0.0677, 0.0150, and 0.0119 records per sq km, respectively. Among these herbaria, FURB also has the largest number of herbarium specimens with photographs available online (4,354 in total), and the goal is to start photographing all specimens before the end of 2014. It also deserves to be mentioned that the collection has 1,417 unique names not found in other herbaria. This is quite a high number despite the fact that, due to the impracticability of this analysis, possible synonyms were not excluded.

Although the flora of Santa Catarina is well documented in the FURB herbarium, recent studies are still reporting new records for the state (Gasper and Sevegnani 2010, Gasper et al. 2012b, Funez and Gasper, unpublished data), suggesting that there are even more species to find.

The Conservation Units are represented at FURB by 2,428 specimens collected in Serra do Itajaí National Park, the largest remaining area of Evergreen Rainforest (Atlantic Rain Forest), 4,771 specimens being collected in other Conservation Units (Table 1).

**Table 1.** Number of specimens from main Conservation Units represented in the herbarium.

Protected area	Species
Morro do Baú Municipal Park	101
Canela Preta Biological Reserve	123
Prima Luna Private Reserve of Natural Patrimony	159
Lagoinha do Leste Municipal Park	165
Caraguatá Private Reserve of Natural Patrimony	194
São Joaquim National Park	211
Bugerkopf Private Reserve of Natural Patrimony	248
Acaraí State Park	260
Lagoa do Peri Municipal Park	283
Dona Francisca Environmental Protection Area	292
Serra do Tabuleiro State Park	340
Rio Vermelho Humboldt Environmental Protection Area	522
São Francisco de Assis Municipal Park	592
Serra do Itajaí National Park	2428

## Taxonomic coverage

The main herbarium collection is composed of vascular plants (Figure 1), representing 4,267 species. Among the vascular plants is the collection of ferns (368 species and 7,904 specimens) and lycophytes (33 species with 456 specimens), which corresponds to 20% of the total specimens and 9,4% of the species. Bryophytes *lato sensu* correspond to 0,2% of the collection. There are 198 families of angiosperms (Figure 2), 33 of ferns, 3 of lycophytes and 6 of gymnosperms. There are 203 plants without identification. The main collections are from Santa Catarina State (Figure 4).

With the implementation of an exchange program and the aim of supporting other collections, the FURB herbarium has donated 19,521 herbarium specimens. The families with more than 500 duplicates donated are: Solanaceae (532 donations), Bromeliaceae (542), Aspleniaceae (612), Piperaceae (769), Orchidaceae (848), Myrtaceae (854), Melastomataceae (1,026), Rubiaceae (1,054), Polypodiaceae (1,156), Fabaceae (1,257), Asteraceae (1,478), and Lauraceae (1,621). Likewise, 3,794 herbarium specimens have already been loaned to perform theses, dissertations, and other taxonomic studies.

It is worth mentioning that there are five types in the collection: Holotypus of *Vriesea rubens* J.G.Silva & A.F.Costa (Bromeliaceae), isotypus of *Croton pygmaeus* L.R.Lima (Euphorbiaceae), paratypus of *Calystegia brummittii* P.P.A.Ferreira & Sim.-Bianch (Convovulaceae), paratypus of *Pleurostachys arcuate* W.W. Thomas, M. Alves & R. Trevis (Cyperaceae), and paratypus of *Sarcoglottis catharinensis* Mancinelli & E.C.Smidt (Orchidaceae).

## Taxonomic ranks

**Kingdom:** Chlorobionta

**Subclass:** Lycopodiidae (lycophytes), Equisetidae, Marattiidae, Ophioglossidae, Polypodiidae, Psilotidae (ferns), Ginkgoidae, Cycadidae, Pinidae, Gnetidae (gymnosperms), and Magnoliidae (angiosperms)

**Lycophyte** families: Isoetaceae (1 specimen/1 species); Lycopodiaceae (326/21) and Selaginellaceae (129/11).

**Fern** families: Anemiaceae (261 specimens/6 families); Aspleniaceae (833/32); Athyriaceae (234/13); Blechnaceae (542/18); Culcitaceae (1/1); Cyatheaceae (425/10); Cystopteridaceae (2/1); Davalliaceae (2/1); Dennstaedtiaceae (70/9); Dicksoniaceae (69/2); Dryopteridaceae (954/43); Equisetaceae (13/3); Gleicheniaceae (99/9); Hemidictyaceae (1/1); Hymenophyllaceae (578/30); Lindsaeaceae (185/8); Lomariopsidaceae (40/6); Lygodiaceae (32/1); Marattiaceae (104/5); Marsileaceae (3/3); Ophioglossaceae (25/4); Osmundaceae (12/2); Parkeriaceae (3/1); Plagiogyriaceae (2/1); Polypodiaceae (2163/60); Psilotaceae (5/1); Pteridaceae (760/54); Saccolomataceae (44/2); Salviniaceae (16/6); Schizaeaceae (25/2); Tectariaceae (68/4); and Thelypteridaceae (330/31).

**Gymnosperm** families: Araucariaceae (17 specimens/3 species); Cupressaceae (59/10); Cycadaceae (6/3); Ginkgoaceae (2/1); Pinaceae (26/8); and Podocarpaceae (40/4).

**Magnoliidae** families: Acanthaceae (384 specimens/35 species); Achatocarpaceae (2/2); Actinidiaceae (1/1); Adoxaceae (15/3); Aizoaceae (3/2); Alismataceae (14/4); Alstroemeriaceae (23/4); Amaranthaceae (185), 23); Amaryllidaceae (51/13); Anacardiaceae (135/13); Annonaceae (271/23); Apiaceae (84/22); Apocynaceae (339/70); Aquifoliaceae (196/8); Araceae (547/35); Araliaceae (123/21); Arecaceae (154/14); Aristolochiaceae (13/6); Asparagaceae (15/6); Asteraceae (2839/426); Balanophoraceae (10/2); Balsaminaceae (7/1); Basellaceae (10/1); Begoniaceae (325/24); Berberidaceae (16/3); Bignoniaceae (271/53); Bixaceae (8/2); Boraginaceae (172/23); Brassicaceae (23/8); Bromeliaceae (1676/85); Burmanniaceae (11/1); Burseraceae (12/1); Cabombaceae (1/1); Cactaceae (356/28); Calceolariaceae (4/3); Calyceraceae (12/2); Campanulaceae (81/19); Canellaceae (17/2); Cannabaceae (65/4); Cannaceae (25/2); Capparaceae (18/3); Caprifoliaceae (32/7); Cardiopteridaceae (13/3); Caricaceae (11/3); Caryocaraceae (4/1); Caryophyllaceae (31/12); Casuarinaceae (8/1); Celastraceae (116/18); Ceratophyllaceae (1/1); Chloranthaceae (37/1); Chrysobalanaceae (44/13); Cleomaceae (5/2); Clethraceae (65/2); Clusiaceae (140/9); Combretaceae (46/14); Commelinaceae (176/19); Connaraceae (11/3); Convolvulaceae (133/33); Costaceae (15/4); Crassulaceae (3/1); Cucurbitaceae (124/26); Cunoniaceae (52/5); Cyclanthaceae (26/1); Cyperaceae (656/109); Dilleniaceae (60/7); Dioscoreaceae (36/10); Droseraceae (2/1); Ebenaceae (11/4); Elaegnaceae (1/1); Elaeocarpaceae (43/5); Ericaceae (69/11); Eriocaulaceae (48/15); Erythroxylaceae (74/14); Escalloniaceae (27/5); Euphorbiaceae (594/83); Fabaceae (1611/323); Fagaceae (8/6); Gentianaceae (29/7); Geraniaceae (3/2); Gesneriaceae (364/25); Goodeniaceae (4/1); Griseliniaceae (18/1); Gunneraceae (8/1); Heliconiaceae (64/3); Hernandiaceae (1/1); Humiriaceae (8/2); Hydrangeaceae (1/1); Hydrocharitaceae (3/1); Hydroleaceae (5/1); Hypericaceae (35/5); Hypoxidaceae (7/1); Icacinaceae (3/1); Iridaceae (97/17); Juglandaceae (2/1); Juncaceae (59/9); Juncaginaceae (1/1); Krameriaceae (3/1); Lacistemataceae (5/3); Lamiaceae (448/70); Lauraceae (921/66); Lecythidaceae (4/2); Lentibulariaceae (15/5); Liliaceae (13/2); Linaceae (1/1); Linderniaceae (3/3); Loasaceae (4/2); Loganiaceae (69/8); Loranthaceae (56/5); Lythraceae (120/21); Magnoliaceae (26/3); Malpighiaceae (255/43); Malvaceae (388/66); Marantaceae (97/10); Marcgraviaceae (33/3); Melastomataceae (1811/157); Meliaceae (368/18); Menispermaceae (41/6); Menyanthaceae (5/1); Molluginaceae (3/1); Monimiaceae (297/12); Moraceae (259/24); Musaceae (5/3); Myristicaceae (16/4); Myrtaceae (1899/257); Nyctaginaceae (132/11); Nymphaeaceae (3/1); Ochnaceae (152/8); Olacaceae (25/2); Oleaceae (15/4); Onagraceae (166/18); Opiliaceae (2/1); Orchidaceae (2611/288); Orobanchaceae (22/6); Oxalidaceae (53/10); Papaveraceae (3/2); Passifloraceae (116/23); Paulowniaceae (7/1); Pentaphylacaceae (9/2); Peraceae (54/2); Phrymaceae (2/1); Phyllanthaceae (72/11); Phytolaccaceae (92/12); Picramniaceae (21/4); Piperaceae (1528/89); Pittosporaceae (1/1); Plantaginaceae (90/18); Platanaceae (2/1); Plumbaginaceae (3/2); Poaceae (641/155); Podostemaceae (3/1); Polygalaceae (93/11); Polygonaceae (110/21); Pontederiaceae (24/9); Portulacaceae (7/6); Primulaceae (427/21); Proteaceae (36/7); Quillajaceae (9/1); Ranunculaceae (25/6); Rapateaceae (3/2); Rhamnaceae (58/11); Rhizophoraceae (7/1); Rosaceae (178/17); Rubiaceae (1779/129); Rutaceae (256/23); Sabiaceae (32/1); Salicaceae (195/21); Santalaceae (59/10); Sapindaceae (435/40); Sapotaceae (100/15); Saxifragaceae (1/1); Schlegeliaceae (7/1); Schoepfiaceae (4/2); Scrophulariaceae (32/3); Simaroubaceae (14/4); Siparunaceae (5/1); Smilacaceae (68/4); Solanaceae (1218/131); Styracaceae (53/4); Symplocaceae (84/15); Talinaceae (15/1); Tamaricaceae (1/1); Theaceae (18/2); Thymelaeaceae (37/3); Trigoniaceae (19/1); Tropaeolaceae (2/1); Typhaceae (8/2); Urticaceae (208/25); Velloziaceae (6/5); Verbenaceae (286/38); Violaceae (47/8); Viscaceae (3/0); Vitaceae (53/8); Vivianiaceae (12/1); Vochysiaceae (28/15); Winteraceae (103/2); Xanthorrhoeaceae (2/1); Xyridaceae (27/11); and Zingiberaceae (27/8).

## Research activities

Herbarium collections have a fundamental importance in biodiversity conservation, exhibiting aspects such as reducing the distribution of a particular species or accumulating information in regard to rare or endangered species (e.g. Schatz 2002; Iganci and Morim 2012), and they often serve as a starting point for conservation (Bridson and Forman et al. 1992; for more information see Pyke and Ehrlich 2010). Currently, many species are described using the herbarium specimens, and this fact according to Bebber et al. (2010), is one of the great frontiersof new species.

To date, the collection has enabled comprehensive floristic studies of the three forest types of Santa Catarina (Gasper et al. 2012a, 2013a, 2013b; Sevegnani et al. 2013a). Phytosociological analyses were conducted by Schorn et al. (2012), Lingner et al. (2013a), and Meyer et al. (2013a). Understory species and regeneration strata were researched by Meyer et al. (2012, 2013b, 2013c). Spatial distribution of vascular plant and fern diversity were analyzed by Gasper and Sevegnani (2010), Gasper et al. (2012b), and Uhlmann et al. (2012). The influence of environmental (geoclimatic) variables on species richness, composition, and distribution in forest remnants was investigated within Evergreen Rainforest by Lingner et al. (2013b), fern species being considered by Gasper et al. (2013c). Spatial patterns of *Dicksonia sellowiana* Hook. (Dicksoniaceae), a threatened species, have been described by Gasper et al. (2011) in a study that was also based on the FURB collection. Finally, the collection has enabled studies on secondary succession and the assessment of the conservation status of the sampled forests (Vibrans et al. 2012c; Sevegnani et al. 2013b, 2013c).

In the future, the phylogenetic information will be provided by the DNA stored in the bank and will be extremely important for taxonomic studies. For this reason it is crucial that incentives for research and the maintenance of herbarium activities continue.

## Current situation and future perspectives

Of the 170 active herbaria listed in the Catalogue of the Brazilian Herbaria (http://www.botanica.org.br/rede_herbarios.php), most have fewer than 50,000 records (80% according to Peixoto et al. 2006). It is estimated that the FURB herbarium will reach this value in two years, and in the meantime must have its registration approved as “Fiel Depositário” (Azevedo 2005; CGEN 2014).

In addition to the novelties promoted by the e-taxonomy, the availability of data online, including the recently added images, allows for the rapid updating of herbariums (Smith and Figueiredo 2009;. Smith et al. 2011), as well as the discovery of gaps in collections (Canhos et al. 2013).

Finally, one of the novelties of the FURB herbarium is the expansion of its fungi collection. This may result from the second cycle IFFSC, with the collection of macrofungi, which requires special care (Wu et al. 2004). The collection of macrofungi in the Herbarium FURB is small at presentand has only 159 specimens, stemmed mainly from sporadic collections of a small number of students.
